# Supplementary material for: Cross-Reactive Antibodies to SARS-CoV-2 and MERS-CoV in Pre-COVID-19 Blood Samples from Sierra Leoneans
Source: Viruses. 2021 Nov 21;13(11):2325. doi: 10.3390/v13112325 (PMC8625389; doi:10.3390/v13112325)
Supplement: Supplementary file 1 [file viruses-13-02325-s001.zip › viruses-1422563- rsupl for pub.pdf]

viruses

MDPI

SUPPLEMENTAL MATERIAL

Cross-reactive antibodies to SARS-CoV-2 and MERS-CoV in pre-COVID-19 blood samples from Sierra Leoneans

Rodrigo Borrega [1,†], Diana K. S. Nelson [2,†], Anatoliy P. Koval [1,†], Nell G. Bond [3,†], Megan L. Heinrich [1,*], Megan M. Rowland [1], Raju Lathigra [1], Duane J. Bush [2], Irina Aimukanova [2], Whitney N. Phinney [2], Sophia A. Koval [1], Andrew R. Hoffmann [3], Allison R. Smither [3], Antoinette R. Bell-Kareem [3], Lilia I. Melnik [3], Kaylynn J. Genemaras [3,4], Karissa Chao [3,4], Patricia Snarski [5,6], Alexandra B. Melton [7], Jaikin E. Harrell [3], Ashley A. Smira [8], Debra H. Elliott [8], Julie A. Rouelle [8], Gilberto Sabino-Santos, Jr. [9,10], Arnaud C. Drouin [11], Mambu Momoh [12,13,14], John Demby Sandi [13], Augustine Goba [13], Robert J. Samuels [13], Lansana Kanneh [13], Michael Gbakie [13], Zoe L. Branco [1], Jeffrey G. Shaffer [15], John S. Schieffelin [8,16], James E. Robinson [8], Dahlene N. Fusco [11], Pardis C. Sabeti [17,18,19,20,21,22], Kristian G. Andersen [23,24], Donald S. Grant [13,14,*], Matthew L. Boisen [2,*], Luis M. Branco [1,*] and Robert F. Garry [1,2,3*]

1 Zalgen Labs, LCC, Germantown, MD 20876 USA rborrega@zalgenlabs.com (R.B.); akoval@zalgenlabs.com (A.P.K.); mrowland@zalgenlabs.com (M.M.R.); rlathigra@zalgenlabs.com (R.L.); sophia.koval@gmail.com (S.A.K.); zbranco@zalgenlabs.com (Z.L.B.)
2 Zalgen Labs, LCC, Broomfield, CO 80045, USA; dnelson@zalgenlabs.com (D.K.S.N.); dbush@zalgenlabs.com (D.J.B.); iaimukanova@zalgenlabs.com (I.A.); wphinney@zalgenlabs.com (W.N.P.)
3 Department of Microbiology and Immunology, School of Medicine, Tulane University, New Orleans, LA 70112, USA; nbond@tulane.edu (N.G.B.); ahoffman@tulane.edu (A.R.H.); asmither@tulane.edu (A.R.S.); abell13@tulane.edu (A.R.B.-K.); lmelnik@tulane.edu (L.I.M.); kgenemar@tulane.edu (K.J.G.); kchao1@tulane.edu (K.C.); jharrel3@tulane.edu (J.E.H.)
4 Bioinnovation Program, Tulane University, New Orleans, LA 70118, USA
5 Heart and Vascular Institute, John W. Deming Department of Medicine, School of Medicine, Tulane University, New Orleans, LA 70112, USA; psnarski@tulane.edu
6 Department of Physiology, School of Medicine, Tulane University, New Orleans, LA 70112, USA
7 Department of Microbiology, Tulane National Primate Research Center, Covington, LA 70433, USA; amelton@tulane.edu
8 Department of Pediatrics, School of Medicine, Tulane University, New Orleans, LA, 70112 USA; areyna@tulane.edu (A.A.S.); dholton@tulane.edu (D.H.E.); jrouelle@tulane.edu (J.A.R.); jschieff@tulane.edu (J.S.S.); jrobinso@tulane.edu (J.E.R.)
9 Department of Tropical Medicine, School of Public Health and Tropical Medicine, Tulane University, New Orleans, LA 70112, USA; gsabino@tulane.edu
10 Centre for Virology Research, Ribeirão Preto Medical School, University of Sao Paulo, Ribeirao Preto 14049900, SP, Brazil
11 Department of Medicine, School of Medicine, Tulane University, New Orleans, LA 70112, USA; adrouin@tulane.edu (A.C.D.); dfusco@tulane.edu (D.N.F.)
12 Eastern Polytechnic Institute, Kenema, Sierra Leone; mmomoh@tulane.edu
13 Viral Hemorrhagic Fever Program, Kenema Government Hospital, Kenema, Sierra Leone; johnatsandi@gmail.com (J.D.S.); augstgoba@yahoo.com (A.G.); robert.j.samuels@vanderbilt.edu (R.J.S.); Lansanakanneh@gmail.com (L.K.); gbakiemichael@gmail.com (M.G.)
14 Ministry of Health and Sanitation, Freetown, Sierra Leone
15 Department of Biostatistics and Data Science, School of Public Health and Tropical Medicine, Tulane University New Orleans, LA 70112, USA; jshaffer@tulane.edu
16 Department of Internal Medicine, School of Medicine, Tulane University, New Orleans, LA, USA
17 Broad Institute of Massachusetts Institute of Technology (MIT) and Harvard, Cambridge, MA 02142, USA; pardis@broadinstitute.org
18 Howard Hughes Medical Institute, Chevy Chase, MD 20815, USA
19 Department of Organismic and Evolutionary Biology, Harvard University, Cambridge, MA 02138, USA
20 Department of Immunology and Infectious Diseases, Harvard T.H. Chan School of Public Health, Harvard University, Boston, MA 02115, USA
21 Department of Medicine, Division of Infectious Diseases, Massachusetts General Hospital, Boston, MA 02114, USA

[22] Massachusetts Consortium on Pathogen Readiness, Boston, MA 02115, USA
[23] Department of Immunology and Microbial Science, Scripps Research, La Jolla, CA 92037, USA; kga1978@gmail.com
[24] Scripps Research Translational Institute, La Jolla, CA 92037, USA
* Correspondence: donkumfel@yahoo.co.uk (D.S.G.); mboisen@zalgenlabs.com (M.L.B.); lbranco@zalgenlabs.com (L.M.B.); rfgarry@tulane.edu (R.F.G.)
† These authors equally contributed to this work.

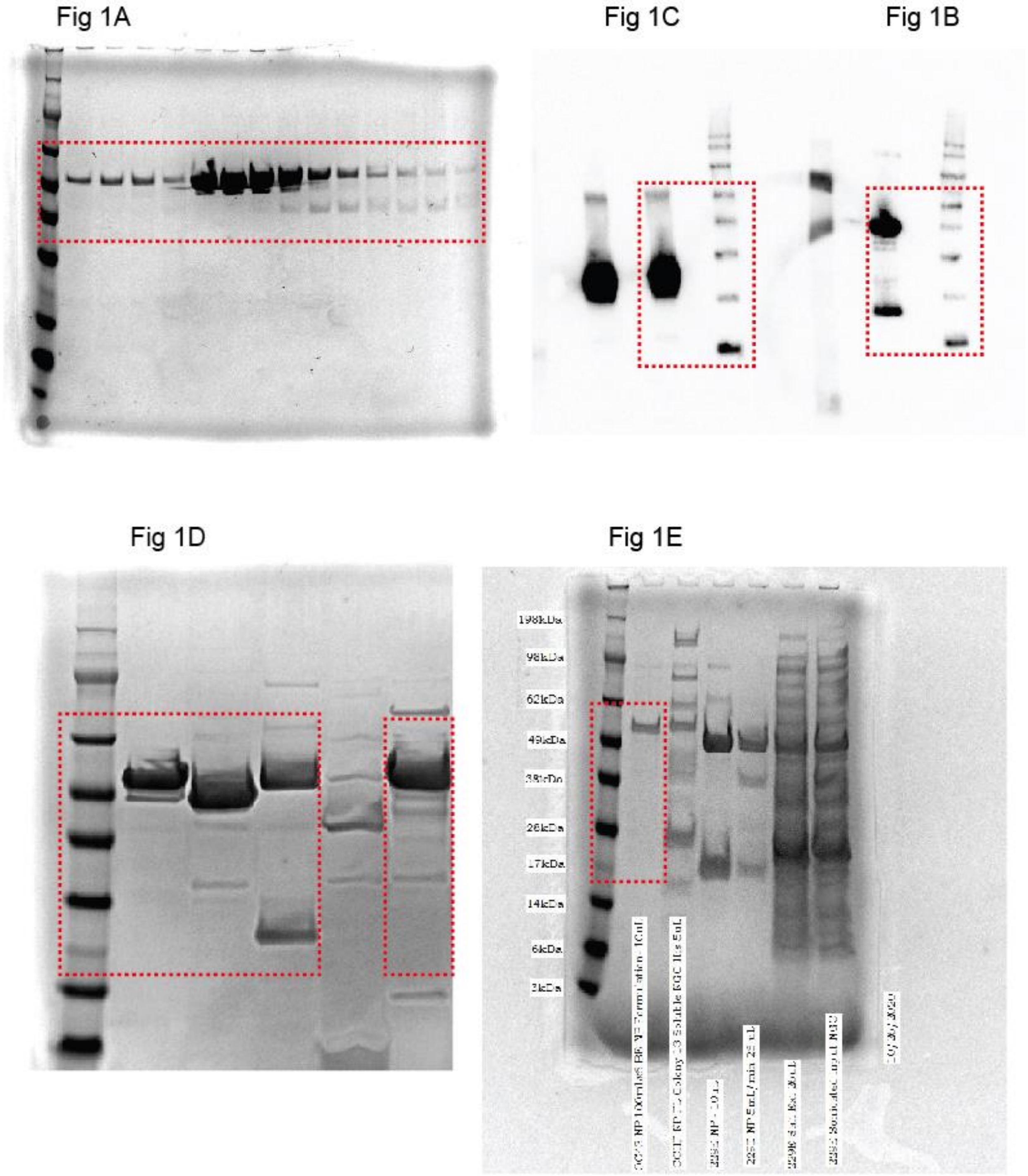

Figure S1. Uncropped gels. The original images of the gels used to assemble Figure 1 are shown. Red dotted square indicate the regions used to assemble the indicated panels.

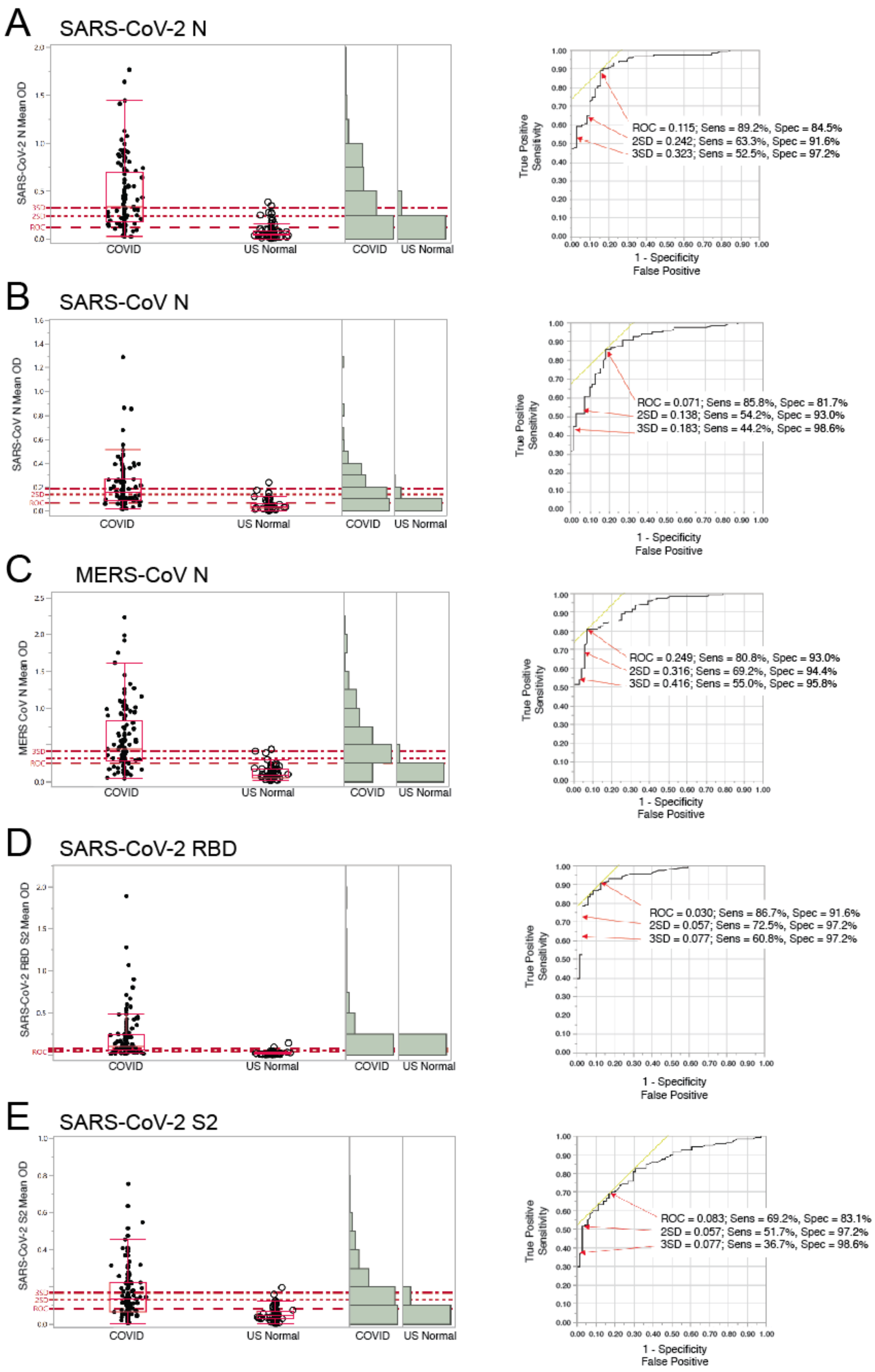

Figure S2. Dot plots and Receiver Operator Characteristic curves. Data used to calculate cut-offs used for coronavirus ELISAs are depicted. Panel A: SARS-CoV-2 N. Panel B: SARS-CoV N. Panel C: MERS-CoV N. Panel D: SARS-CoV-2 RBD. Panel E: SARS-CovV-2 S2 Spike subunit.

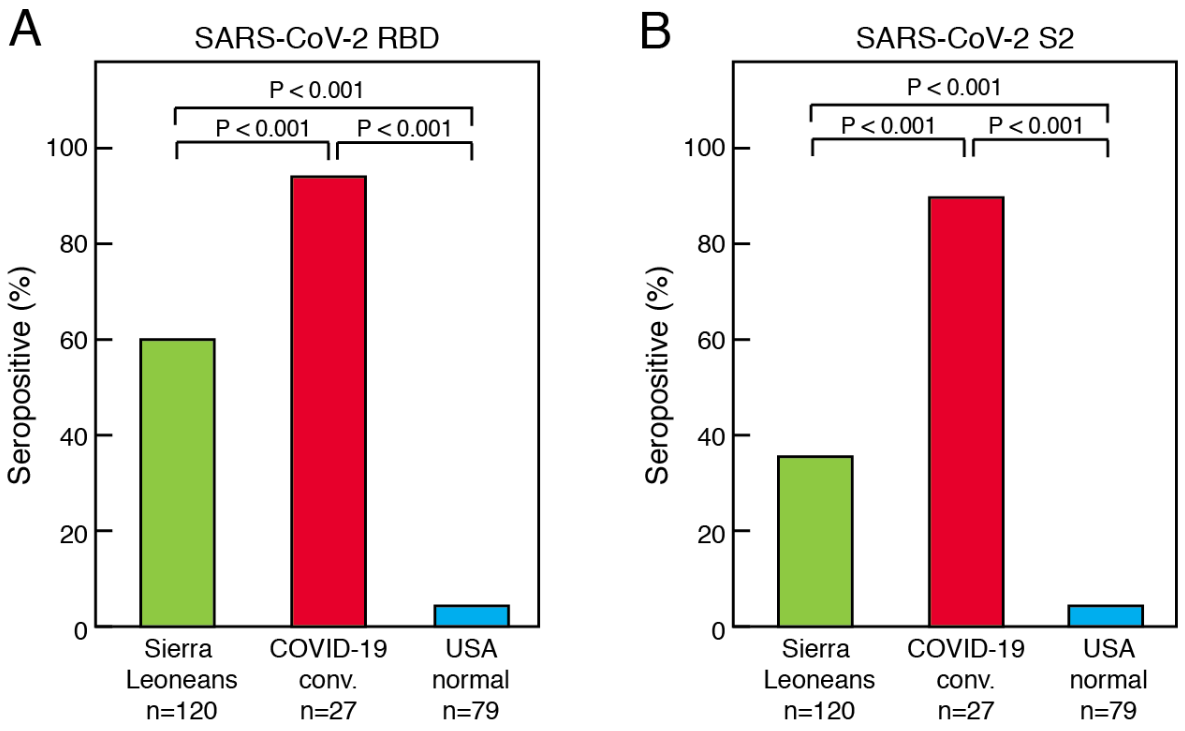

Figure S3. Serological responses to coronavirus antigens by Sierra Leoneans, COVID-19 subjects and United States normal blood donors. Percent positive results for the indicated coronavirus proteins for the three cohorts are shown. Panel A: SARS-CoV-2 RBD. Panel B: SARS-CoV-2 S2 Spike subunit.

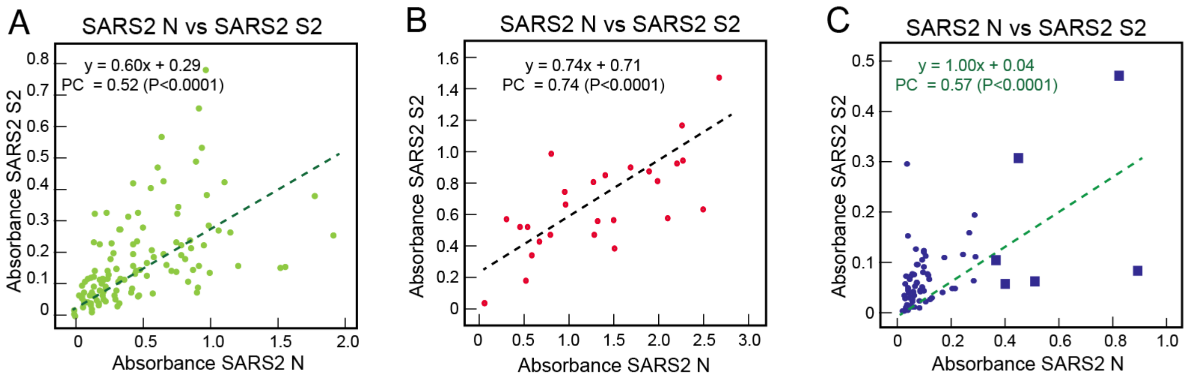

Figure S4. Correlations for SARS-CoV-2 N versus SARS-CoV2 S2 subunit of spike in Sierra Leoneans, COVID subjects, and healthy blood donors. Panel A: Pre-pandemic Sierra Leoneans. Panel B: COVID-19 subjects. Panel C; United States normal blood donors.

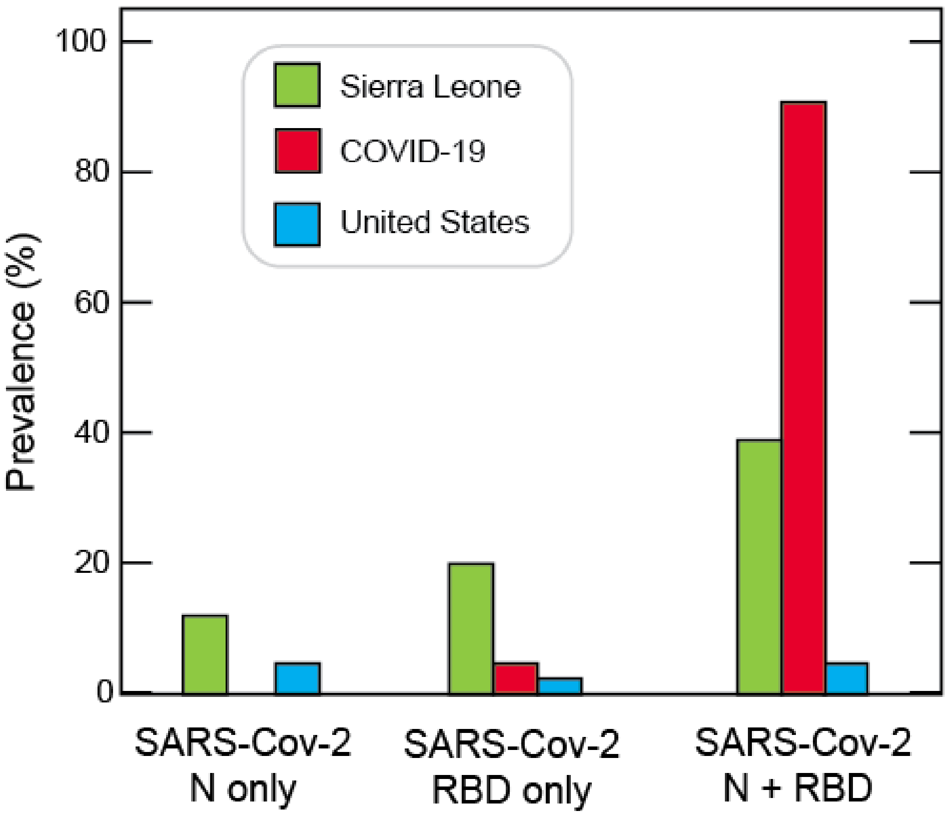

100
80
60
40
20
0
Prevalence (%)
Sierra Leone
COVID-19
United States
SARS-Cov-2
N only
SARS-Cov-2
RBD only
SARS-Cov-2
N + RBD

Figure S5. Reactivities to N or S or both in Sierra Leoneans, COVID survivors, and healthy blood donors.

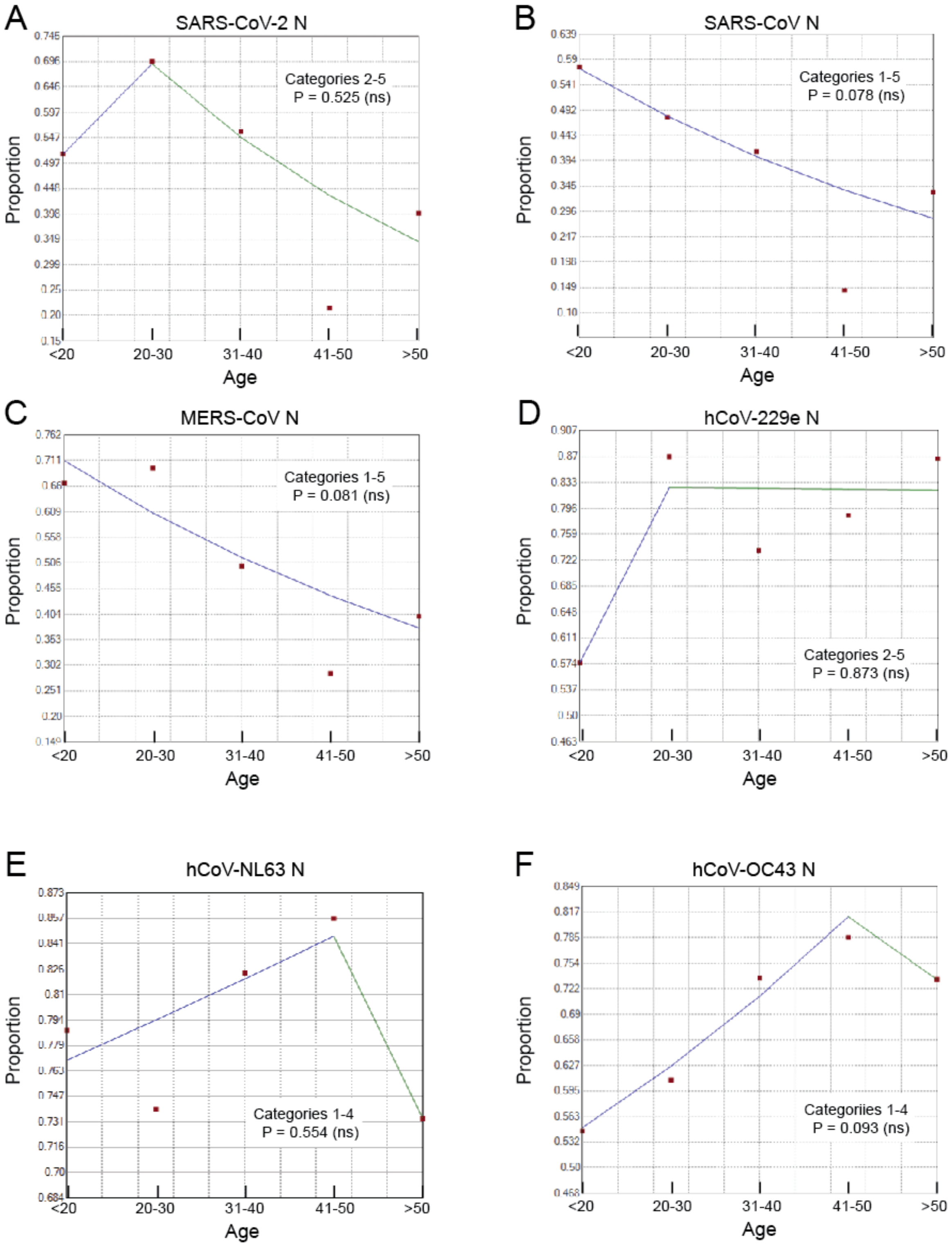

Figure S6. Coronavirus reactivity by age in Sierra Leoneans. JoinPoint regression analyses for seroreactivity to the indicated coronavirus N was performed. Panel A: SARS-CoV-2 N. Panel B: SARS-CoV N. Panel C: MERS-CoV N. Panel D: hCoV-229E. Panel E: hCoV-NL63. Panel F: hCoV-OC43.

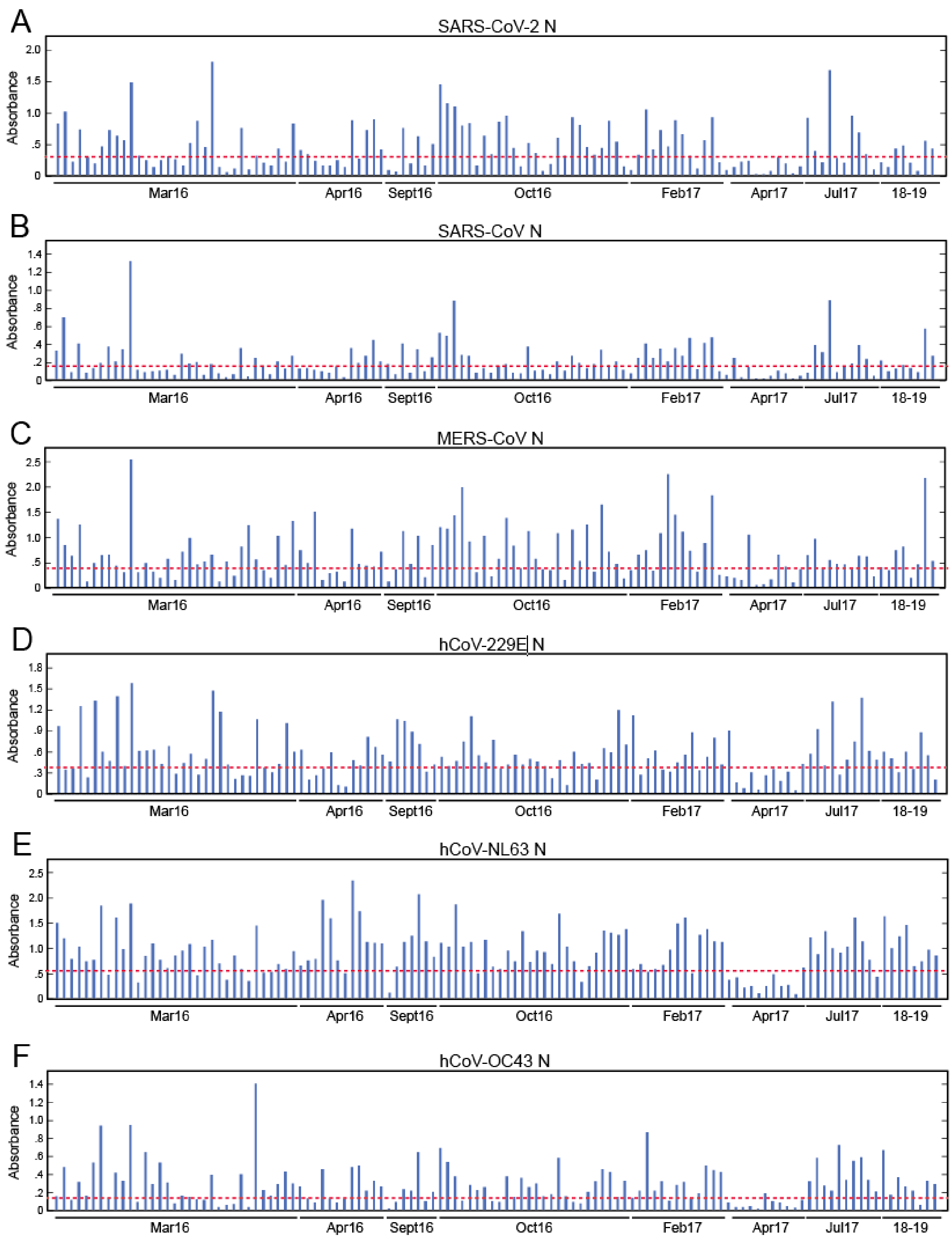

Figure S7. Reactivity to different coronavirus N by time of sampling. Seroreactivities in Sierra Leonean samples are plotted by time of collection. Panel A: SARS-CoV-2 N. Panel B: SARS-CoV N. Panel C: MERS-CoV N. Panel D: hCoV-229E. Panel E: hCoV-NL63. Panel F: hCoV-OC43.

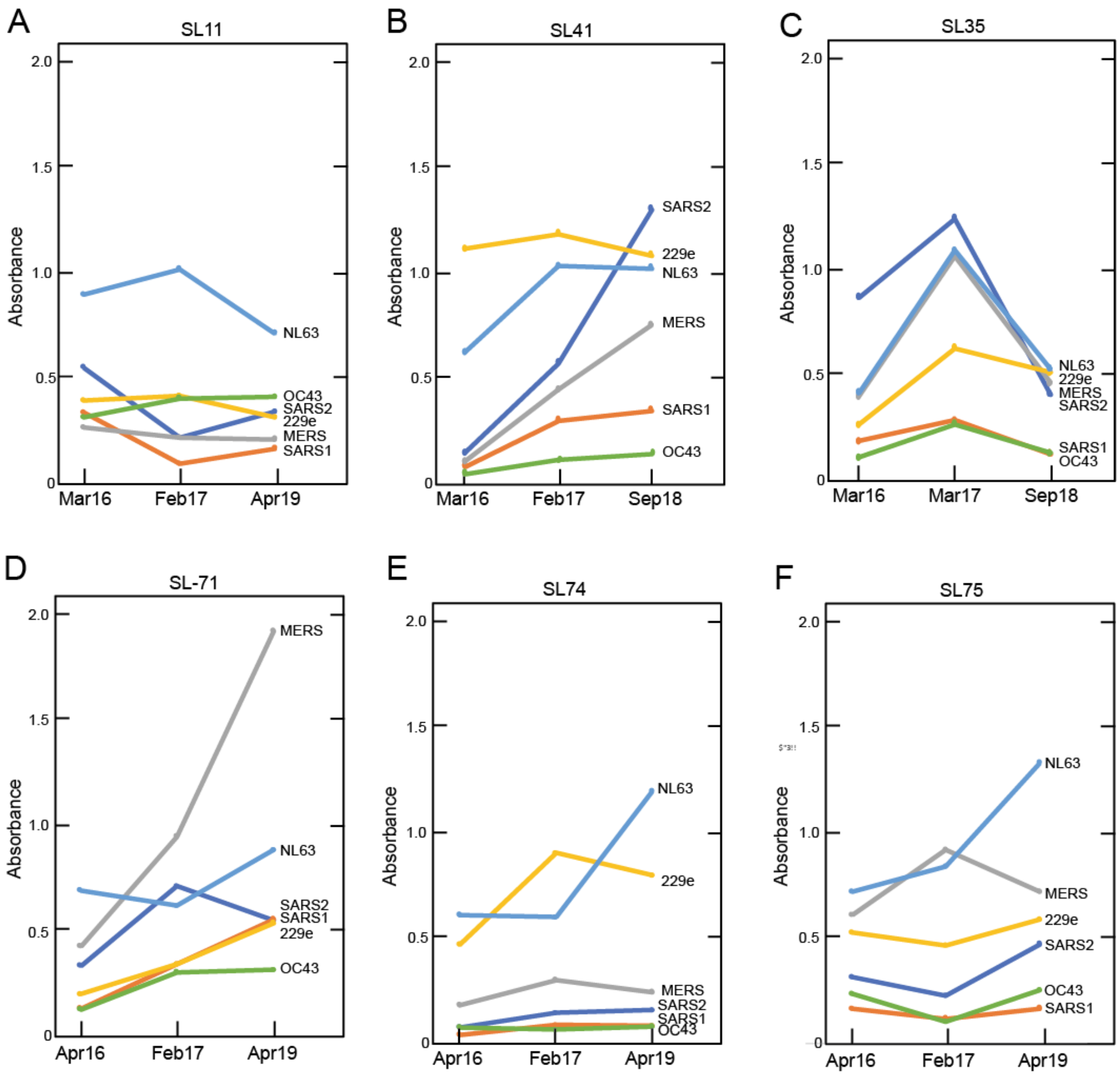

Figure S8. Changes in reactivity to coronaviruses in individual Sierra Leonean subjects over time. Variation in absorbance values for seroreactivity to N of SARS-CoV-2, SARS-CV, MERS-CoV, hCoV-229E, hCoV-NL63 and hCoV-OC43. Panel A: SL11. Pabel B: SL41. Panel C: SL35. Panel D: SL71. Panel E: SL74. Panel F: SL75.

Table S1. Reactivity of antibodies in samples from Sierra Leoneans collected prior to the CoVID-19 pandemic to coronavirus proteins.[1]

SL No.	Draw Date	SARS-2 Mean OD	SARS1 Mean OD	MERS Mean OD	229E Mean OD	NL63 Mean OD	OC43 Mean OD	SC2-RBD Mean OD	SC2-S2 Mean OD	age	sex
1	2-Oct-16	0.163	0.096	0.172	0.312	1.030	0.100	0.038	0.316	7.5	F
2	2-Oct-16	0.489	0.248	0.741	0.411	0.750	0.192	0.600	0.273	8.5	F
3	27-Sep-16	0.092	0.173	0.102	0.451	0.116	0.026	0.062	0.049	10.0	F
4	21-Feb-17	0.309	0.455	0.639	0.858	0.461	0.124	0.316	0.148	10.4	F
5	2-Oct-16	1.411	0.513	1.047	0.519	1.003	0.659	0.225	0.152	11.0	F
6	2-Oct-16	1.126	0.476	1.026	0.391	0.932	0.508	0.212	0.156	11.5	F
7	14-Mar-16	0.115	0.062	0.206	0.215	0.782	0.064	0.115	0.117	13.0	F
8	8-Apr-17	0.133	0.237	0.166	0.166	0.387	0.035	0.089	0.065	13.0	F
9	14-Mar-16	0.740	0.347	0.719	0.263	0.537	0.384	0.331	0.190	14.0	F
10	14-Apr-16	0.247	0.147	0.273	0.126	0.695	0.085	0.093	0.142	15.0	F
11	12-Mar-16	0.857	0.194	0.401	0.268	0.419	0.117	0.074	0.091	15.0	F
12	25-Apr-19	0.469	0.166	0.718	0.585	1.326	0.251	0.141	0.144	15.0	F
13	15-Feb-17	0.321	0.238	0.574	0.274	0.631	0.210	0.137	0.109	15.8	F
14	8-Apr-17	0.225	0.031	0.124	0.087	0.210	0.036	0.486	0.056	16.0	F
15	9-Mar-16	0.223	0.089	0.547	0.363	0.719	0.109	0.106	0.115	18.0	F
16	2-Oct-16	1.070	0.855	1.260	0.462	1.690	0.355	0.803	0.259	18.5	F
17	30-Sep-16	0.742	0.395	0.985	1.010	1.025	0.221	0.170	0.219	18.6	F
18	10-Mar-16	0.710	0.362	0.572	0.456	0.437	0.137	0.056	0.139	20.0	F
19	12-Mar-16	0.447	0.053	0.445	0.487	0.932	0.110	0.030	0.231	20.0	F
20	1-Apr-16	0.407	0.122	0.656	0.617	0.599	0.251	0.040	0.077	20.0	F
21	2-Oct-16	0.773	0.274	1.753	0.728	0.924	0.103	0.157	0.178	20.0	F
22	8-Apr-17	0.236	0.138	0.925	0.300	0.232	0.049	0.032	0.094	20.5	F
23	20-Jul-17	0.895	0.076	0.565	0.560	1.095	0.306	0.027	0.220	21.4	F
24	20-Jul-17	0.390	0.379	0.846	0.899	0.807	0.555	0.394	0.306	21.4	F
25	2-Oct-16	0.824	0.267	0.800	1.081	1.015	0.269	0.164	0.144	25.0	F
26	10-Mar-16	0.624	0.203	0.373	1.359	1.447	0.396	0.076	0.413	28.0	F
27	9-Mar-16	0.725	0.392	1.102	1.218	0.925	0.298	0.452	0.338	30.0	F
28	12-Mar-16	1.767	0.172	0.578	1.433	1.055	0.372	0.265	0.250	30.0	F
29	2-Oct-16	0.161	0.078	0.259	0.536	0.462	0.215	0.033	0.062	30.0	F
30	2-Oct-16	0.624	0.136	0.903	0.441	1.063	0.245	0.902	0.193	30.5	F
31	11-Mar-16	0.302	0.108	0.501	0.670	0.553	0.288	0.156	0.055	31.0	F
32	8-Apr-17	0.025	0.014	0.041	0.066	0.107	0.021	0.023	0.009	31.0	F
33	20-Jul-17	0.216	0.303	0.338	0.401	1.210	0.265	0.425	0.110	31.4	F
34	16-Feb-17	0.416	0.241	0.292	0.612	0.543	0.213	0.233	0.087	31.9	F
35	12-Mar-16	0.141	0.074	0.100	1.140	0.635	0.037	0.012	0.025	32.0	F
36	15-Apr-16	0.134	0.030	0.105	0.109	0.464	0.128	0.023	0.038	32.0	F
37	20-Feb-17	0.859	0.347	1.271	0.443	1.355	0.271	0.482	0.156	32.9	F
38	20-Feb-17	0.642	0.261	0.980	0.552	1.452	0.302	0.244	0.161	32.9	F
39	10-Mar-16	0.551	0.336	0.266	0.394	0.898	0.313	0.065	0.072	33.0	F

40	25-Jul-17	0.932	0.177	0.327	0.723	1.448	0.525	0.071	0.137	33.4	F
41	28-Feb-17	0.217	0.092	0.217	0.415	1.019	0.404	0.091	0.065	34.0	F
42	9-Mar-16	0.291	0.082	0.096	0.235	0.675	0.154	1.069	0.196	34.0	F
43	25-Apr-19	0.198	0.133	0.168	0.352	0.585	0.212	0.209	0.083	34.1	F
44	14-Mar-16	0.105	0.041	1.083	0.250	0.317	0.040	0.017	0.047	35.0	F
45	8-Apr-17	0.023	0.019	0.052	0.262	0.232	0.183	0.029	0.022	35.00	F
46	2-Oct-16	0.331	0.076	0.193	0.752	0.574	0.096	0.027	0.044	35.0	F
47	2-Oct-16	0.844	0.158	0.501	0.364	0.537	0.093	0.421	0.475	35.5	F
48	2-Oct-16	0.930	0.169	1.215	0.408	0.870	0.355	1.281	0.280	35.5	F
49	10-Mar-16	1.445	1.291	2.235	1.532	1.707	0.897	0.670	0.154	36.0	F
50	11-Mar-16	0.257	0.060	0.128	0.287	0.778	0.074	0.033	0.128	36.0	F
51	25-Jul-17	0.675	0.380	0.554	1.334	1.035	0.560	0.298	0.142	36.4	F
52	2-Oct-16	0.431	0.082	0.729	0.547	0.672	0.144	0.154	0.132	37.5	F
53	15-Mar-16	0.161	0.064	0.160	0.298	0.484	0.155	0.082	0.143	39.0	F
54	8-Apr-17	0.083	0.049	0.135	0.353	0.447	0.095	0.011	0.030	39.0	F
55	2-Oct-16	0.148	0.071	0.335	0.412	1.214	0.341	0.022	0.065	40.0	F
56	20-Jul-17	1.639	0.864	0.474	1.273	0.899	0.208	0.168	0.371	40.4	F
57	2-Oct-16	0.514	0.360	0.992	0.485	0.658	0.245	1.892	0.127	40.5	F
58	25-Jul-17	0.337	0.229	0.539	0.601	0.706	0.323	0.209	0.117	41.4	F
59	14-Mar-17	0.097	0.060	0.193	0.886	0.351	0.082	0.037	0.107	42.0	F
60	20-Jul-17	0.278	0.088	0.410	0.276	0.829	0.690	0.054	0.225	42.3	F
61	30-Mar-16	0.223	0.123	0.386	0.980	0.541	0.408	0.119	0.078	43.0	F
62	30-Sep-16	0.190	0.079	0.410	0.866	1.140	0.209	0.140	0.044	45.6	F
63	2-Oct-16	0.361	0.099	0.501	0.450	0.867	0.281	0.047	0.113	46.5	F
64	30-Jul-17	0.099	0.046	0.187	0.480	0.393	0.204	0.026	0.045	50.4	F
65	2-Oct-16	0.080	0.108	0.319	0.393	0.842	0.147	0.135	0.098	50.5	F
66	5-Dec-18	0.422	0.123	0.652	0.302	1.122	0.349	0.053	0.224	50.5	F
67	14-Mar-16	0.308	0.239	0.492	1.039	1.311	1.331	0.136	0.148	51.0	F
68	2-Oct-16	0.177	0.061	0.301	0.226	0.622	0.169	0.102	0.051	52.5	F
69	2-Oct-16	0.587	0.199	0.956	0.469	1.534	0.549	0.080	0.455	52.7	F
70	30-Mar-16	0.805	0.261	1.158	0.584	0.852	0.283	0.262	0.234	10.0	M
71	13-Apr-16	0.335	0.132	0.427	0.200	0.691	0.128	0.555	0.268	12.0	M
72	25-Apr-16	0.268	0.187	0.411	0.398	1.566	0.469	0.187	0.176	12.0	M
73	24-Feb-17	0.556	0.401	0.771	0.514	1.246	0.473	0.164	0.182	12.8	M
74	16-Feb-17	0.709	0.340	0.945	0.341	0.617	0.303	0.529	0.229	12.8	M
75	13-Apr-16	0.234	0.109	1.330	0.267	0.721	0.080	0.060	0.049	13.0	M
76	21-Apr-16	0.860	0.352	1.030	0.471	2.117	0.453	0.435	0.636	13.0	M
77	16-Feb-17	0.459	0.205	1.988	0.316	0.876	0.101	0.106	0.066	13.8	M
78	27-Feb-17	0.906	0.463	1.616	0.780	1.034	0.425	0.368	0.754	13.9	M
79	25-Apr-19	0.079	0.085	0.402	0.856	0.671	0.058	0.063	0.057	14.2	M
80	15-Mar-16	0.419	0.204	0.901	0.415	0.622	0.276	0.114	0.156	15.0	M
81	25-Apr-19	0.548	0.553	1.918	0.536	0.882	0.314	0.357	0.203	15.0	M
82	2-Oct-16	0.316	0.101	0.120	0.129	0.934	0.153	0.027	0.034	16.5	M
83	10-Mar-16	0.245	0.088	0.430	0.608	0.761	0.612	0.037	0.319	18.0	M
84	10-Mar-16	0.308	0.108	0.265	0.597	0.290	0.087	0.143	0.080	18.0	M

85	8-Apr-17	0.295	0.105	0.570	0.180	0.237	0.084	0.053	0.068	19.0	M
86	7-Oct-16	0.143	0.107	0.154	0.687	1.249	0.313	0.060	0.076	20.1	M
87	2-Oct-16	0.912	0.260	1.008	0.583	0.679	0.088	0.206	0.372	20.5	M
88	11-Mar-16	0.159	0.289	0.627	0.430	0.867	0.155	0.112	0.123	22.0	M
89	11-Mar-16	0.514	0.180	0.869	0.559	0.985	0.139	0.109	0.164	25.0	M
90	7-Sep-16	0.880	0.432	0.362	0.661	1.006	0.312	0.572	0.516	26.0	M
91	8-Apr-17	0.189	0.074	0.363	0.316	0.256	0.046	0.069	0.038	26.0	M
92	8-Apr-17	0.032	0.016	0.088	0.059	0.097	0.028	0.013	0.004	27.0	M
93	2-Oct-16	0.792	0.184	0.459	0.422	0.305	0.072	0.228	0.109	27.0	M
94	3-Sep-18	0.218	0.213	0.347	0.586	1.482	0.635	0.081	0.118	29.4	M
95	2-Oct-16	0.451	0.130	1.103	0.426	0.587	0.195	0.082	0.065	30.0	M
96	7-Sep-19	0.421	0.261	0.458	0.199	0.773	0.275	0.437	0.193	32.0	M
97	8-Mar-16	0.811	0.317	1.197	0.938	1.367	0.152	0.253	0.210	33.0	M
98	9-Mar-16	0.196	0.129	0.431	1.301	0.696	0.498	0.108	0.223	34.0	M
99	2-Oct-16	0.328	0.173	0.271	0.207	0.833	0.309	0.336	0.266	34.9	M
100	7-Sep-16	0.418	0.199	0.629	0.548	0.997	0.255	0.715	0.407	35.0	M
101	9-Mar-16	0.452	0.185	0.563	0.591	1.672	0.885	0.055	0.320	35.0	M
102	2-Oct-16	0.440	0.328	1.447	0.640	1.221	0.434	0.246	0.140	38.5	M
103	8-Apr-17	0.143	0.048	0.309	0.419	0.565	0.116	0.018	0.016	41.0	M
104	10-Mar-16	0.137	0.095	0.281	0.617	0.997	0.276	0.056	0.122	42.0	M
105	12-Mar-16	0.057	0.036	0.447	0.409	0.347	0.063	0.018	0.066	42.0	M
106	3-Sep-18	0.140	0.097	0.306	0.502	0.903	0.162	0.062	0.041	44.5	M
107	13-Apr-16	0.157	0.091	0.126	0.359	1.770	0.432	0.014	0.094	45.0	M
108	8-Mar-16	0.992	0.676	0.739	0.346	1.084	0.454	0.055	0.264	45.0	M
109	21-Feb-17	0.119	0.118	0.281	0.327	1.145	0.179	0.015	0.046	45.9	M
110	10-Mar-16	0.249	0.104	0.166	0.424	0.698	0.498	0.056	0.117	47.0	M
111	30-Sep-16	0.614	0.336	0.902	0.697	1.870	0.610	0.129	0.548	50.6	M
112	20-Jul-17	0.199	0.142	0.399	0.482	0.932	0.321	0.085	0.114	52.3	M
113	13-Apr-16	0.156	0.082	0.249	0.581	1.440	0.124	0.041	0.228	53.2	M
114	28-Sep-16	0.076	0.067	0.311	1.037	0.571	0.091	0.073	0.064	53.5	M
115	10-Feb-17	0.094	0.074	0.298	1.089	0.532	0.136	0.093	0.077	53.9	M
116	15-Feb-17	1.034	0.396	0.655	0.499	0.482	0.818	0.167	0.410	54.0	M
117	2-Oct-16	0.851	0.162	0.630	0.580	1.184	0.407	0.071	0.075	55.5	M
118	2-Oct-16	0.536	0.205	0.409	1.167	1.143	0.140	0.233	0.393	55.5	M
119	14-Mar-16	0.208	0.158	0.301	0.374	0.468	0.220	0.050	0.102	60.0	M
120	26-Apr-16	0.714	0.266	0.372	0.793	1.016	0.212	0.058	0.317	NR	NR

1. Values in cells that are filled pink are positive.
2. NR= not recorded

Table S2. Reactivity of antibodies in samples from United States normal blood donors to coronavirus proteins.[1]

	SARS-2	SARS1	MERS	229E	NL63	OC43	SC2-RBD	SC2-S2
C19 No.	Mean OD	Mean OD	Mean OD	Mean OD	Mean OD	Mean OD	Mean OD	Mean OD
1	0.978	0.772	0.082	1.021	0.507	0.136	0.936	0.632
2	1.445	0.824	0.098	0.913	0.382	0.063	2.144	0.813
3	2.058	1.242	0.431	0.457	0.439	0.089	1.812	0.776
4	0.447	0.289	0.084	1.130	0.418	0.137	0.171	0.492
5	2.350	1.504	0.166	1.323	1.891	0.097	1.673	0.902
6	0.027	0.037	0.041	0.302	0.153	0.103	0.037	0.026
7	2.593	1.559	0.546	1.459	1.844	0.150	2.007	0.599
8	0.530	0.267	0.172	0.847	0.750	0.366	0.405	0.493
9	0.801	0.491	0.144	0.160	0.258	0.337	0.747	0.444
10	2.287	1.479	0.084	0.197	0.192	0.083	2.014	0.881
11	1.312	0.711	0.063	0.816	0.303	0.265	1.227	0.445
12	0.673	0.365	0.072	1.120	0.332	0.046	0.868	0.403
13	2.347	1.438	0.114	1.040	1.438	0.066	1.893	1.113
14	1.960	1.167	0.070	0.780	0.236	0.087	1.818	0.833
15	1.351	0.817	0.072	1.134	0.507	0.186	0.991	0.530
16	1.541	0.969	0.091	0.783	1.199	0.226	1.520	0.535
17	0.967	0.634	0.042	1.171	0.073	0.030	0.953	0.710
18	0.290	0.153	0.074	0.922	0.554	0.055	0.650	0.543
19	2.179	1.303	0.695	0.886	1.916	0.904	0.785	0.548
20	1.306	0.815	0.188	0.137	0.128	0.036	1.006	0.768
21	0.031	0.028	0.083	0.473	0.802	0.225	0.006	0.029
22	0.581	0.306	0.048	0.511	0.353	0.084	0.145	0.322
23	1.738	0.995	0.131	1.577	1.320	0.170	0.973	0.858
24	0.516	0.253	0.129	0.352	0.469	0.134	0.294	0.165
25	0.809	0.365	0.111	0.356	0.955	0.267	0.238	0.943
26	2.774	1.745	0.329	1.078	1.807	0.246	1.151	1.410
27	1.548	0.842	0.181	0.916	0.597	0.255	1.432	0.365

1. Values in cells that are filled with pink are positive.

Table S3. Reactivity of antibodies in samples from United States normal blood donors to coronavirus proteins.[1]

	SARS-2	SARS1	MERS	229E	NL63	OC43	SC2-RBD	SC2-S2
US No.	Mean OD	Mean OD	Mean OD	Mean OD	Mean OD	Mean OD	Mean OD	Mean OD
P8-01	0.066	0.026	0.085	0.149	0.312	0.104	0.003	0.009
P8-02	0.087	0.068	0.053	0.514	0.332	0.190	0.008	0.114
P8-03	0.227	0.120	0.229	0.420	0.503	0.534	0.015	0.117
P8-04	0.054	0.073	0.965	0.938	1.647	0.378	0.009	0.097
P8-05	0.051	0.057	0.068	0.256	0.254	0.185	0.008	0.128
P8-06	0.081	0.149	0.072	0.170	0.480	0.223	0.010	0.043
P8-07	0.869	0.354	0.118	0.581	0.397	0.104	0.012	0.085
P8-08	0.151	0.103	0.194	0.494	0.442	0.161	0.016	0.041
P8-09	0.069	0.033	0.098	0.300	0.826	0.424	0.016	0.042
P8-10	0.016	0.012	0.070	0.250	1.184	0.360	0.009	0.073
P8-11	0.348	0.090	0.394	1.256	1.522	0.201	0.094	0.107
P8-12	0.036	0.018	0.116	0.296	0.402	0.246	0.003	0.011
P8-13	0.803	0.040	0.052	1.318	0.684	0.518	0.010	0.476
P8-14	0.096	0.238	0.091	0.602	1.014	0.561	0.012	0.079
P8-15	0.494	0.111	0.099	1.377	1.149	0.246	0.984	0.062
P8-16	0.045	0.049	0.441	0.307	0.346	0.024	0.032	0.077
P8-17	0.090	0.040	0.086	0.988	0.531	0.575	0.035	0.023
P8-18	0.046	0.048	0.298	0.699	0.492	0.558	0.009	0.035
P8-19	0.038	0.016	0.067	0.370	0.684	0.093	0.007	0.022
P8-20	0.098	0.071	0.185	1.140	0.728	0.342	0.007	0.075
P8-21	0.196	0.028	0.066	0.343	0.469	0.050	0.010	0.048
P8-22	0.266	0.149	0.109	0.666	0.455	0.313	0.023	0.064
P8-23	0.271	0.174	0.441	0.327	1.093	0.415	0.142	0.197
P8-24	0.188	0.063	0.120	0.666	0.914	0.118	0.014	0.049
P8-25	0.060	0.066	0.120	0.419	0.669	0.263	0.012	0.097
P8-26	0.029	0.155	0.190	0.538	0.611		0.023	0.030
P8-27	0.023	0.158	0.970	0.578	0.889	1.002	0.023	0.155
P8-28	0.037	0.045	0.126	0.834	0.328	0.136	0.030	0.034
P8-29	0.025	0.025	0.069	0.513	1.266	0.113	0.016	0.039
P8-30	0.040	0.054	0.100	0.704	0.796	0.179	0.019	0.040
P8-31	0.251	0.091	0.094	1.225	1.098	0.323	0.030	0.161
P8-32	0.066	0.038	0.055	0.911	0.712	0.315	0.010	0.024
P8-33	0.157	0.119	0.106	0.891	1.331	0.176	0.045	0.111
P8-34	0.384	0.064	0.182	0.580	0.547	0.125	0.047	0.058
P8-35	0.103	0.085	0.223	1.160	0.697	0.133	0.018	0.067
P8-36	0.076	0.040	0.222	0.471	0.531	0.059	0.012	0.106
P8-37	0.078	0.037	0.141	0.762	0.224	0.042	0.016	0.082
P8-38	0.429	0.173	0.214	1.154	0.482	0.138	0.088	0.312
P8-39	0.040	0.105	0.089	0.994	0.329	0.331	0.016	0.078

P8-40	0.039	0.035	0.417	0.486	0.466	0.262	0.009	0.027
P9-01	0.082	0.078	0.076	1.144	0.940	1.031	0.009	0.125
P9-02	0.017	0.012	0.033	0.288	0.043	0.011	0.006	0.010
P9-03	0.042	0.021	0.052	0.092	0.261	0.073	0.021	0.042

P9-04	0.038	0.020	0.047	0.087	0.241	0.068	0.016	0.037
P9-05	0.011	0.002	0.025	0.009	0.070	0.005	0.001	0.003
P9-06	0.019	0.014	0.015	0.052	0.065	0.021	0.004	0.007
P9-07	0.082	0.056	0.247	0.337	0.336	0.173	0.028	0.092
P9-08	0.056	0.042	0.188	0.284	0.284	0.136	0.021	0.059
P9-09	0.054	0.045	0.208	0.270	0.293	0.144	0.020	0.061
P9-10	0.018	0.021	0.021	0.175	0.106	0.049	0.006	0.016
P9-11	0.024	0.018	0.021	0.100	0.084	0.030	0.007	0.014
P9-12	0.036	0.029	0.174	0.203	0.208	0.093	0.011	0.030
P9-13	0.037	0.031	0.171	0.188	0.203	0.100	0.013	0.034
P9-14	0.035	0.027	0.151	0.198	0.187	0.100	0.014	0.030
P9-15	0.043	0.033	0.179	0.232	0.222	0.119	0.017	0.039
P9-16	0.024	0.011	0.033	0.059	0.326	0.084	0.018	0.049
P9-17	0.029	0.015	0.041	0.081	0.397	0.108	0.023	0.067
P9-18	0.023	0.011	0.031	0.061	0.310	0.081	0.016	0.050
P9-19	0.274	0.049	0.051	0.200	0.604	0.118	0.014	0.112
P9-20	0.024	0.013	0.031	0.053	0.301	0.076	0.017	0.045
P9-21	0.033	0.029	0.088	0.241	0.648	0.140	0.022	0.073
P9-22	0.021	0.015	0.086	0.420	0.702	0.072	0.006	0.005
P9-23	0.080	0.438	0.135	0.144	0.861	0.123	0.063	0.121
P9-36	0.050	0.010	0.024	0.109	0.821	1.157	0.008	0.054
P9-25	0.018	0.033	0.030	0.935	0.785	0.068	0.011	0.300
P9-26	0.012	0.021	0.049	0.068	0.310	0.078	0.007	0.029
P9-28	0.003	0.007	0.024	0.134	0.556	0.289	0.004	0.003
P9-29	0.017	0.019	0.068	0.289	0.228	0.279	0.012	0.011
P9-30	0.014	0.021	0.039	0.728	0.460	0.263	0.013	0.058
P9-31	0.035	0.021	0.047	0.174	0.275	0.048	0.010	0.037
P9-32	0.075	0.042	0.055	0.523	0.695	0.286	0.006	0.051
P9-27	0.008	0.010	0.030	0.442	0.211	0.139	0.009	0.035
P9-34	0.045	0.016	0.088	0.219	0.238	0.031	0.006	0.012
P9-35	0.045	0.015	0.101	0.201	0.266	0.030	0.003	0.011
P9-36	0.074	0.019	0.036	0.168	0.605	0.966	0.012	0.050
P9-37	0.070	0.017	0.033	0.159	0.593	0.888	0.011	0.046
P9-38	0.111	0.062	0.155	0.626	0.814	0.078	0.010	0.029
P9-39	0.103	0.057	0.137	0.640	0.815	0.081	0.010	0.027
P9-40	0.079	0.018	0.028	0.186	0.646	0.887	0.014	0.046

1. Values in cells that are filled with pink are positive.

Table S4. Distribution of samples from Sierra Leoneans with positive serology for various coronaviruses by age.

		SARS-2		SARS1		MERS		229E		NL63		OC43		Total
Age Category		n	%	n	%	n	%	n	%	n	%	n	%	n
1.00	<20	17.00	0.52	19.00	0.58	22.00	0.67	19.00	0.58	26.00	0.79	18.00	0.55	33.00
2.00	20-30	16.00	0.70	11.00	0.48	16.00	0.70	20.00	0.87	17.00	0.74	14.00	0.61	23.00
3.00	31-40	19.00	0.56	14.00	0.41	17.00	0.50	25.00	0.74	28.00	0.82	25.00	0.74	34.00
4.00	41-49	3.00	0.21	2.00	0.14	4.00	0.29	11.00	0.79	12.00	0.86	11.00	0.79	14.00
5.00	≥50	6.00	0.40	5.00	0.33	6.00	0.40	13.00	0.87	11.00	0.73	11.00	0.73	15.00

Table S5. Distribution of samples from Sierra Leoneans with positive serology for various coronaviruses by sex.

			SARS1		MERS		229E		NL63		OC43		Total
	n	%	n	%	n	%	n	%	n	%	n	%	n
M	25	0.500	23	0.460	28	0.560	38	0.760	41	0.820	33	0.660	50
F	36	0.522	28	0.406	37	0.536	50	0.725	53	0.768	46	0.667	69

Table S6. Neutralization of SARS-CoV2 or MERS-CoV pseudoviruses by antibodies from blood collected from Sierra Leoneans prior to the COVID-19 pandemic.

Pseudovirus	Number of samples	Positive samples (%)
SARS-CoV-2	47	12 (25.5)
MERS-CoV	30	10 (33.3)
